# Reduction of the Six-Minute Walk Distance in Children with Sickle Cell Disease Is Correlated with Silent Infarct: Results from a Cross-Sectional Evaluation in a Single Center in Belgium

**DOI:** 10.1371/journal.pone.0108922

**Published:** 2014-10-02

**Authors:** Laurence Dedeken, Rudy Chapusette, Phu Quoc Lê, Catherine Heijmans, Christine Devalck, Sophie Huybrechts, France Ziereisen, Laurence Hanssens, Laurence Rozen, Denis Noubouossie, Malou Ngalula Mujinga, Alina Ferster

**Affiliations:** 1 Department of Hematology and Oncology, Hôpital Universitaire des Enfants Reine Fabiola, Université Libre de Bruxelles, Brussels, Belgium; 2 Department of Radiology, Hôpital Universitaire des Enfants Reine Fabiola, Université Libre de Bruxelles, Brussels, Belgium; 3 Department of Pneumology and Allergology, Hôpital Universitaire des Enfants Reine Fabiola, Université Libre de Bruxelles, Brussels, Belgium; 4 Laboratory of Hematology, Centre Hospitalier Universitaire-Brugmann/Hôpital Universitaire des Enfants Reine Fabiola, Université Libre de Bruxelles, Brussels, Belgium; Brighton and Sussex Medical School, United Kingdom

## Abstract

**Background:**

The 6-minute walk test (6MWT) is used in adults and children affected by a wide range of chronic diseases to evaluate their sub-maximal exercise capacity. It reflects the global response of various physiological systems in a situation simulating a daily life activity.

**Methods:**

We analyzed factors affecting the 6MWT in 46 Sickle Cell Disease children. Forty-two were treated with hydroxyurea (HU). Patients with normal test (>80% of the age-standardized predicted value) were compared to patients with abnormal test (≤80%). Baseline hematological values, clinical events, cerebrovascular disease, cardio-pulmonary parameters and disease-modifying treatment were compared according to the performance of the test.

**Results:**

Among the 46 patients, 14 had an abnormal 6MWT. In univariate analysis, both groups were similar for biological and clinical data. Six of the 14 patients with an abnormal 6MWT had silent infarct (SI) compared to 6/32 with a normal test (*P* = 0.09). When excluding chronically transfused patients, 4 of the 11 patients with an abnormal 6MWT had SI compared to 1/26 (*P* = 0.02). Baseline pulse oximetry was normal in both groups but slightly lower in patients with abnormal 6MWT (*P* = 0.02). No patient presented exercise-induced desaturation. In multivariate analysis, the only factor associated with abnormal 6MWT was the presence of SI (*P* = 0.045).

**Conclusions:**

In our cohort of 46 patients characterized by high exposure rate to HU and by the absence of severe cardiopulmonary disease, the sole factor independently associated with 6MWT was the presence of SI. The lower exercise capacity of children with SI may reflect some subclinical neurological impairment as they do not differ by hemoglobin level or cardiopulmonary parameters.

## Introduction

Sickle cell disease (SCD) caused primarily by a mutation in the β-globin chain of hemoglobin has variable phenotype. A majority of patients suffer from repeated episodes of pain in the context of vaso-occlusive crisis (VOC). They will also experience acute chest syndrome (ACS) and some will develop overt stroke. Long term complications include chronic organ damage, like pulmonary hypertension, osteonecrosis, renal failure which may lead to end-stage organ failure. However, morbidity, frequency of crisis and degree of anemia vary from individual to individuals.

First introduced for adults suffering from pulmonary or cardiovascular conditions [1], [2], the six-minute walk test (6MWT) is progressively implemented among pediatric patients to evaluate their sub-maximal functional exercise capacity. During 6 minutes, the participant has to walk as fast as possible and the walking distance (6MWD) is compared to a validated and standardized formula as previously reported by Geiger [3]. This submaximal test reflects the global response of various physiological systems (respiratory, cardiovascular, nervous, metabolic and musculoskeletal system) in a situation simulating a daily life activity. The few studies having explored the 6MWT in SCD children have shown that low hemoglobin level, low fetal hemoglobin and low red cell deformability were independent predictors of reduced 6MWT performance [4], [5]. Low hemoglobin and increased hemolysis also predict low oxygen saturation at baseline and after exercise [6]–[8].

Our study aimed to explore the submaximal exercise capacity and to analyze factors affecting the 6MWT and the 6MWD in the cohort of SCD children and adolescents followed at the Hôpital Universitaire des Enfants Reine Fabiola, Brussels, Belgium.

## Design and Methods

From September 2011 6MWT was incorporated in our standard annual evaluation to measure if the daily fonctional capacity was altered. All patients with SCD above 6 years of age attending the outpatient clinic for their annual evaluation between September 2011 and March 2012 were invited to particiapte in this study. Patients were recruited on a consecutive basis; no attempt was made to select them by known or perceived risk factors. The study was conducted in accordance with the Declaration of Helsinki and was approved by the Ethics Committee of the Hôpital Universitaire des Enfants Reine Fabiola, Brussels (February 28, 2012). As the 6MWT was incorporated in our standard annual evaluation from September 2011, for this observational study, only verbal consent was obtained from patients and parents as approved by the Ethics Committee. Inclusion criteria were: i. presence of a severe sickle cell syndrome (all phenotype included), ii. regular follow-up since at least 3 years, iii. agreement to perform 6MWT, iv. steady state defined by no hospitalisation or emergency visit for pain crisis, ACS, stroke, priapism, splenic sequestration or other SCD related acute complications in the last month, v. no blood transfusion for an acute event in the previous 3 months. Exclusion criteria were the presence of severe motor, orthopedic or cognitive disability (precluding instructions understanding), at the time of the inclusion compromising their participation to the test. The 6MWT was performed according to the guidelines of the American Thoracic Society [9]. The age-standardized predicted value of the 6MWD was established by Geiger [3] and takes into account age, sex and height. The 6MWT was considered as normal if the 6MWD was more than 80% of the age-standardized predicted value, moderately decreased between 60–80%, and severely altered if less than 60%. Children were separated in 2 groups: group A if the performance was more than 80% and group B if the performance was equal or less than 80%. Pulse oximetry measurements as well as blood pressure, cardiac and respiratory rates were measured before and after the 6MWT. Pulmonary function testing was added to the annual evaluation. Baseline hematological values, clinical events, cerebrovascular disease, cardio-pulmonary parameters (evaluated by pulmonary function tests, blood pressure, heart rate, systolic function and tricuspid regurgitant jet velocity, TRV) and disease-modifying treatment were compared according to the performance of the test.

Statistical analyses were computed using version 5 of the software Graphpad Prism (Graphpad Software Inc, San Diego, USA). As several variables showed a skewed distribution, all data were presented as median and range. Variables were compared between groups in univariate analyses using the Mann Whitney test for continuous variables and the Fisher exact test for proportions. All variables with p value below 0.2 were included in multivariate logistic regression models to identify the variables independently associated with abnormal 6MWT or the presence of silent infarct. Multivariate analyses were computed using XLSTAT software (Addinosoft, Paris, France). All p values were 2-sided, and a value ≤0.05 indicated statistical significance.

## Results

From September 2011 to March 2012, 46 patients (20 boys and 26 girls) with a median age of 12 years were enrolled and only one with hip osteonecrosis requiring walking stick was excluded. Thirty-nine were HbSS, 4 HbSβ°, 2 HbSC and 1 HbSβ+. Forthy-two patients were treated with HU mainly for recurrent VOC and/or ACS (74%), 9 patients were on chronic exchange transfusion program (2 for stroke prevention, 1 for abnormal TRV and 6 for persistent recurrent VOC and/or ACS despite HU given at maximal tolerated dose). Thirty-two patients had a normal 6MWT (Group A) and 14 an abnormal test (Group B). Only one patient had a severely altered test. These 2 groups were similar for age, sex, genotype and history of VOC or ACS as well as for receiving disease-modifying treatment, either hydroxyurea (HU) or chronic transfusion (Table 1). The median 6MWD was 603 meters (range: 450–720 m) in group A and 458 meters (range: 270–498 m) in group B (*P*<0.001). The analysis performed when excluding chronically transfused patients shows a median 6MWD of 603 meters (range: 450–720 m) in group A and 465 meters (range 270–498 m) in group B (*P*<0.001). The proportion of patients with normal or conditional transcranial doppler echography was also similar in both groups. At the time of the study, no patient had cerebral velocities >200 cm/sec. Only one had experienced a stroke. Silent infarct (SI) on routine cerebral magnetic resonance imaging was found in 42.9% in group B versus only 19.4% in group A (*P* = 0.09) and in 36.4% in group B versus 3.9% in group A when patients chronically transfused were excluded (*P* = 0.02). Cardio-pulmonary parameters were identical in both groups. Severe FEV1/VC ratio reduction and severe TLC reduction less than 60% of the predicted value were observed only in 2 patients in each group. Only one patient had a TRV >2.5 m/sec. Baseline pulse oximetry was normal in both groups but slightly lower in patients with abnormal 6MWT (in the whole cohort as well as in not transfused patients). Pulse oximetry after the 6MWT was similar in both groups. No patient had a saturation reduction ≥3% after the 6MWT compared to baseline value. Biological parameters were not statistically different between both groups. In multivariate analysis, the only factor associated with abnormal 6MWT was the presence of silent infarct (*P* = 0.045). To better assess the role of SI on the 6MWT, data from patients with SI (N = 12) were compared to those without SI (N = 34) (Table 2). The median 6MWD was 503 meters (range: 270–720 m) in patients with SI and 585 meters (range: 390–720 m) in those without SI (*P* = 0.02). Excluding chronically transfused patients, the median 6MWD was 438 meters (range: 270–540 m) in patients with SI and 579 meters (range: 450–720 m) in those without SI (*P* = 0.003). In univariate analyses, patients with SI had significantly reduced 6MWT performance although they were similar for age, sex, previous ACS or painful crisis, hemolytic parameters (reticulocytes counts, LDH) and basal hemoglobin level. Since low total hemoglobin concentration was not found in this series to be significantly correlated with reduced 6MWT performance, we decided to compare our population with the cohort of Waltz in which none of the children was under hydroxyurea treatment [4]. For this comparison, only patients with HbSS or HbSβ° and not chronically transfused (N = 37) were considered (Table 3). Our patients were marginally older, they had a significantly better 6MWD, higher BMI, higher total Hb concentration and higher HbF percentage.

**Table 1:** Characteristics of patients with normal (Group A) and abnormal (Group B) 6MWT; results represent median (range) unless otherwise indicated.

	WHOLE COHORT			UNTRANSFUSED PATIENTS		
	Group A	Group B	p value	Group A	Group B	p value
Number of patients	32	14		26	11	
Age, years	13 (6.1–19.7)	11.5 (8.5–18.0)	0.29	13 (6.1–19.7)	10.4 (8.5–18.0)	0.38
Male gender, n (%)	15 (46.9%)	5 (35.7%)	0.48	9 (34.6%)	4 (36.4%)	1.00
BMI (kg/m^2^)	18.5 (12.7–26.0)	19.6 (14.9–28.9)	0.19	19.8 (14.9–28.9)	18.1 (12.7–26.0)	0.25
African central origin, n (%)	24 (75.0%)	8 (57.1%)	0.23	20 (76.9%)	6 (54.6%)	0.24
HbSS/Sβ0, n (%)	31 (96.6%)	12 (85.7%)	0.16	25 (96.2%)	9 (81.8%)	0.21
G6PD deficiency*	3 (9.7%)	1 (7.7%)	0.83	2 (7.7%)	0	1.00
Number of VOC	1 (1–10)	0 (0–9)	0.58	1 (0–10)	0 (0–9)	0.92
History of ≥2 ACS, n (%)	20 (64.5%)	6 (42.9%)	0.17	14 (53.9%)	4 (36.4%)	0.48
Dactylitis, n (%)	7 (35.0%)	3 (21.4%)	0.78	6 (23.1%)	2 (18.2%)	1.00
Splenic sequestration, n (%)	10 (31.3%)	3 (21.4%)	0.50	8 (30.8%)	2 (18.2%)	0.69
Osteonecrosis femoral head/hip, n (%)	4 (12.5%)	2 (14.3%)	0.87	3 (11.5%)	2 (18.2%)	0.62
Overt Stroke, n (%)	0	1 (7.1%)	0.13	0	0	NA
Silent infarct, n (%)	6 (19.4%)	6 (42.9%)	0.09	1 (3.9%)	4 (36.4%)	0.02
Severe Infections, n (%)	8 (25.0%)	2 (14.3%)	0.39	7 (26.9%)	1 (9.1%)	0.39
Hydroxyurea treatment, n (%)	30 (93.7%)**	12 (85.7%)**	0.37	25 (96.2%)	10 (90.9%)	0.51
Chronic tranfusion program, n (%)	6 (18.8%)	3 (21.4%)	0.83	0	0	NA
Transcranial doppler velocity <170 cm/s, n (%)	30 (96.8%)	11 (84.6%)	0.14	25 (96.2%)	10 (90.9%)	0.51
FEV1/VC (%)	88 (73–134)	95 (78–123)	0.79	93 (73–134)	92 (78–123)	1.00
TLC (% PV)	81 (56–104)	80 (66–114)	0.86	81 (58–104)	77 (66–89)	0.62
SaO2 before 6MWT, (%)	100 (96–100)	98 (97–100)	0.02	100 (96–100)	99 (97–100)	0.046
SaO2 ≤98% after 6MWT, n (%)	7 (22.0%)	4 (28.6%)*	0.72	6 (23.1%)	4 (36.4%)*	0.44
Haemoglobin (g/dl)	9.0 (6.7–12.2)	9.1 (7.6–10.5)	0.99	8.8 (6.7–12.2)	9 (7.6–10.5)	0.93
MCV (fL)	90.4 (64.0–111.7)	92.2 (75.5–115.0)	0.99	94.7 (64.0–111.7)	92.3 (81.0–115.0)	0.82
Hemoglobin fœtal (% Hb total)	13.4 (0.2–27.8)	13.7 (1–18)	0.71	14.8 (4.1–27.8)	14.5 (1.8–18.0)	0.53
Platelets (10^3^/µL)	441 (153–618)	457 (197–1036)	0.42	431 (153–618)	389 (197–1036)	0.99
Reticulocytes (10^3^/µL)	341 (120–491)	277 (155–511)	0.30	318 (120–466)	273 (155–511)	0.54
Polynuclear Neutrophils (10^3^/µL)	4.4 (2.0–7.9)	4.4 (1.4–8.4)	0.84	4.1 (2.0–5.5)	4.2 (1.4–5.7)	0.64
LDH (UI/L)	830 (382–1394)	936 (405–1399)	0.68	831 (382–1394)	927 (406–1400)	0.56

* Missing data for 2 patients.

** In combination with chronic transfusion in 6 patients in group A and in 3 patients in group B.

BMI, Body Mass Index; G6PD, Glucose 6 Phosphate Dehydrogenase; VOC, Vaso-Occulsive Crisis having required an hospitalisation during the last 4 years; ACS, Acute Chest Syndrome; Severe infections: septicemia, osteomyelitis, meningitis; FEV, Forced Expiratory Volume in 1 second; VC, Vital Capacity; TLC, Total Lung Capacity; % PV, Per Cent of the predicted value; 6MWT, 6-Minute Walk Test; MCV, Mean Cell Volume; LDH, Lactate Dehyrogenase.

**Table 2:** 6MWT performance, hematological and clinical factors according to the presence of silent infarct; results are median (range) unless otherwise indicated.

	WHOLE COHORT			UNTRANSFUSED PATIENTS		
	Silent Infarct	No Silent Infarct	p value	Silent Infarct	No Silent Infarct	p value
Number of patients	12	34		5	32	
Age (years)	11.8 (9.2–16.2)	13.0 (6.1–19.7)	0.37	13 (9.2–16.2)	13 (6.1–19.7)	0.86
Male gender, n (%)	7 (58.3%)	13 (38.2%)	0.23	1 (20%)	12 (37.5%)	0.64
6MWT (% PV)	74.9 (63.8–86.9)	86.0 (79.1–92.1)	0.04	64.0 (44.7–84.2)	86.0 (66.3–105.2)	0.003
Haemoglobin (g/dl)	9.7 (8.3–10.5)	8.8 (6.7–12.2)	0.06	9.0 (8.3–10.5)	8.8 (6.7–12.2)	0.45
Haemoglobin fœtal (% Hb total)	2.5 (0.2–16.0)	15.0 (0.2–27.8)	<0.001	11.7 (1.8–24.0)	15.0 (4.1–27.8)	0.09
MVC (fL)	86.3 (75.5–98.0)	94.7 (64.0–115.0)	0.009	86.3 (81.0–98.0)	94.7 (64.0–115.0)	0.21
Polynuclear Neutrophils (10^3^/µL)	6.9 (2.5–8.4)	4.2 (1.4–5.7)	0.01	2.8 (2.5–5.5)	4.2 (1.5–5.7)	0.42
Reticulocytes (10^3^/µL)	273 (133–511)	329 (120–491)	0.55	273 (155–511)	311 (120–466)	0.74
LDH (UI/L)	945 (382–1092)	825 (406–1400)	0.83	927 (406–1026)	831 (382–1400)	0.91
AST (UI/L)	42 (23–46)	36 (18–66)	0.85	42 (20–46)	36 (18–66)	0.64
HbSS/Sβ0, n (%)	11 (91.7%)	32 (94.1%)	0.77	4 (80%)	30 (93.8%)	0.36
ACS	11 (91.7%)	27 (79.4%)	0.42	4 (80%)	25 (78.2%)	1.00
VOC	1 (0–9)	1 (0–10)	0.62	0 (0–9)	1 (0–10)	0.56
Hydroxyurea treatment, n (%)	9 (75.0%)	33 (97.1%)	0.02	4 (80%)	31 (96.9%)	0.56
Chronic tranfusion program, n (%)	7 (58.3%)	2 (5.9%)	<0.001	0	0	NA

6MWT, 6-Minute Walk Test; %PV = per cent of the predicted value; MCV, Mean Cell Volume; LDH, Lactate Dehyrogenase; AST, Aspartate Transaminase; ACS, Acute Chest Syndrome; VOC, Vaso-Occulsive Crisis having required an hospitalisation during the last 4 years.

**Table 3:** Comparison of baseline characteristics and clinical data from Brussels and Waltz series; all values represent mean ± SD unless otherwise indicated.

Study authors	Current Study	Waltz et al.	p value
Number of patients	37*	42	
Age (years)	13.1±3.3	11.7±2.4	0.03
Patients treated with HU	35 (94.6%)	0	<0.0001
6MWD (m)	551±92	491±64	0.001
Percentage of predicted distance (%)	83.2±12.1	74.5±10.0	<0.001
BMI (kg/m2)	19.1±3.8	16.8±2.3	0.002
SpO2 (%)	100 (96–100)**	97.7±2.5	NA
Hemoglobin (g/dl)	8.9±1.04	8.0±1.3	0.001
MCV (fL)	93.1±10.5	79.9±7.7	<0.0001
Hemoglobin fœtal (% Hb total)	15±6.3	8.2±6.4	<0.0001

* Transfused patients were excluded for this comparison. Only HbSS/Sβ^0^ phenotypes were compared.

** Values of SpO2 in our series were not gaussian and are expressed in median with range.

HU, Hydroxyurea; 6MWD, 6 minutes walk distance; BMI, Body Mass; SpO2, Arterial Hemoglobin Oxygen Saturation; MCV, Mean Cell Volume; NA, not applicable.

## Discussion

The 6MWT is a sub-maximal exercise test used in adults and children affected by a wide range of chronic diseases to evaluate their exercise capacity. In adults affected by sickle cell anemia, the reduction of the 6MWD reflects mainly pulmonary hypertension and might be a useful tool to monitor therapeutic intervention [10], [11]. However, other non-cardiopulmonary factors such as chronic pain, osteopenia and hip osteonecrosis may also influence the 6MWD [12]. In children and adolescents, factors affecting the 6MWD are not well established. In this cross-sectional study, the majority of children with SCD had a normal 6MWT whereas in other studies, the majority of patients had an abnormal test [4] or lower walk distance than what we observed [5], [8], [13]–[15]. In contrast to other series, our population is characterized by high treatment rate with HU [4] and also by the absence of severe cardiopulmonary disease. Indeed, only one patient in our study (2.1%) had elevated TRV as compared to 11% in the series of Minniti et al [5]. The proportion of patients receiving transfusion may have contributed to higher 6MWD compared to other series. Nevertheless, when transfused patients are excluded, the 6MWD remains also higher and might be due to significantly higher proportion of our patients treated with HU [4], [8]. History of VOC, ACS, altered lung function testing and overt stroke were not correlated in our population with a lower performance measured during the 6MWT. This lack of any correlation might be due to the small size of our cohort although in larger published series [4], [5], [8], [16], no correlation between 6MWT and history of VOC, ACS or impaired lung function was found either. Waltz et al recently demonstrated the implication of blood rheology on the 6MWT performance with low level of red cell deformability, high level of anemia and low HbF level as independent predictors of a reduced 6MWD [4]. Campbell et al have explored the relationship between the exercise performance and oxygen saturation changes during the 6MWT and hemolytic parameters. In a population of 391 patients having similar age compared to our cohort, but differing by HU prescription rate (144/391) [8], low hemoglobin concentration and high level of hemolysis were the sole independent predictors of low steady state saturation and exercise-induced reduction of saturation. However, the 6MWD was not affected by the value of the saturation at baseline. Of interest, none of our patient presented exercise-induced reduction of saturation though our population is similar for age and genotype but differ in the proportion of patients under HU and with a TRV >2.5 m/s. The effect of HU on pulmonary pressure is still a matter of debate. The beneficial effect obtained by lowering the parameters of hemolysis and increasing hemoglobin level might be counterbalanced by increased HbF level and reduced oxygen delivery to tissue [17]. However HU seems to prevent the development of pulmonary hypertension since the longitudinal follow-up of a cohort of SCD adults indicated that HU reduced the raise of TRV observed with time [18]. The current view of the physiopathology of SCD attributes the clinical complications either to vaso-occlusion (VOC, osteonecrosis, ACS) or hemolysis-endothelial dysfunction associated with proliferative vasculopathy and dysregulated vasomotor function, including leg ulcers, priapism, pulmonary hypertension and possibly non-hemorrhagic stroke [19]–[21]. The association between anemia, hemolysis, hypoxemia at baseline or after exercise and/or the 6MWT performance suggests that desaturation is linked more to hemolysis-endothelial dysfunction than to vaso-occlusion [5]–[8], [13]. Moreover hemolysis estimated by high hemolytic component predicted decline in 6MWD and TRV increase overtime [13]. The mechanisms to explain the association between hemolysis and decreased oxygen saturation are not well understood, but it is hypothesized to represent a manifestation of endothelial dysfunction with dysregulated vasomotor function associated with reduced nitric oxide (NO) bioavailability and responsible for impaired oxygen exchanges. The link between anemia, markers of hemolysis, hypoxemia and the 6MWT performance could not be found in our small series characterized by higher hemoglobin level, higher HbF, probably lower hemolysis, and normal TRV (except in one patient), when compared to other pediatric series [4], [5], [13].

Surprisingly the only independent factor predicting an abnormal 6MWT in our population was SI. The reduced 6MWT performance found in patients with SI is a new observation and we believe that it may reflect some subclinical motor or sensitive impairment since it cannot be explained by the severity of anemia, cardio-pulmonary dysfunction or previous clinical events. Increased fetal hemoglobin level modulates the phenotype of sickle cell anemia by inhibiting HbS polymerization [22], [23]. Attempt to increase HbF levels might play a role in some mechanisms implicated in the development of cerebral vasculopathy in children with SCD such as improving anemia, reducing intravascular hemolysis and NO bioavailability. Recent data issued from the walk-PHaSST study in adults showed that the severity of hemolysis is lower with hydroxyurea therapy, high hemoglobin F level, and concomitant alpha-thalassemia [24]. Nevertheless in the large study of Debaun [25], no association between HbF level and increased risk of SI was found while risks factors for developing SI were low total hemoglobin level, male gender and high systolic blood pressure. Silent infarct seems to be linked more to hemolysis than to vaso-occlusion. The beneficial effect of HU to prevent brain damage and SI remains to be demonstrated. In this series with only one high TRV patient, the sole factor which was independently linked to the 6MWT was the presence of SI. We cannot rule out that minimal cardio-pulmonary disease not detected by our screening test might be responsible for decrease 6MWT in our patients. However, the lower exercise capacity of children with silent stroke may reflect some subclinical neurological impairment and this finding should be confirmed in a larger cohort of patients.
